# Toward a Tiered Model to Share Clinical Trial Data and Samples in Precision Oncology

**DOI:** 10.3389/fmed.2018.00006

**Published:** 2018-01-29

**Authors:** Stefanie Broes, Denis Lacombe, Michiel Verlinden, Isabelle Huys

**Affiliations:** ^1^European Organisation for Research and Treatment of Cancer, Brussels, Belgium; ^2^Department of Pharmaceutical and Pharmacological Sciences, KU Leuven, Leuven, Belgium

**Keywords:** data sharing, precision medicine, oncology, clinical research, biobanking

## Abstract

The recent revolution in science and technology applied to medical research has left in its wake a trial of biomedical data and human samples; however, its opportunities remain largely unfulfilled due to a number of legal, ethical, financial, strategic, and technical barriers. Precision oncology has been at the vanguard to leverage this potential of “Big data” and samples into meaningful solutions for patients, considering the need for new drug development approaches in this area (due to high costs, late-stage failures, and the molecular diversity of cancer). To harness the potential of the vast quantities of data and samples currently fragmented across databases and biobanks, it is critical to engage all stakeholders and share data and samples across research institutes. Here, we identified two general types of sharing strategies. First, open access models, characterized by the absence of any review panel or decision maker, and second controlled access model where some form of control is exercised by either the donor (i.e., patient), the data provider (i.e., initial organization), or an independent party. Further, we theoretically describe and provide examples of nine different strategies focused on greater sharing of patient data and material. These models provide varying levels of control, access to various data and/or samples, and different types of relationship between the donor, data provider, and data requester. We propose a tiered model to share clinical data and samples that takes into account privacy issues and respects sponsors’ legitimate interests. Its implementation would contribute to maximize the value of existing datasets, enabling unraveling the complexity of tumor biology, identify novel biomarkers, and re-direct treatment strategies better, ultimately to help patients with cancer.

## Introduction

Cancer still figures among the leading cause of death and diseases worldwide with approximately 14 million new cases diagnosed in 2012 (1). Historically, the hallmark of cancer treatment consisted of nonspecific cytotoxic agents alone or in combination with radiotherapy and/or surgery. In the past few years, clinical cancer research has seen a remarkable evolution, whereby many new and promising, specific or targeted treatment options including precision medicines and immune-oncology drugs complement the more traditional therapeutic arsenal (2, 3). Precision oncology makes use of the presence of predictive biomarkers that identify patient subpopulations that are likely to show a response to a therapy (4). Oncology, with its genetically driven disease etiology, has typically been at the forefront of this precision medicine revolution.

The oncology market may well be expanding (5, 6); however, stakeholders are confronted with an increasing number of challenges as a consequence of the shift toward precision oncology. Drug developers for instance, acknowledge that research and development (R&D) of precision oncology therapeutics puts the more conventional drug development models under stress (7). The gold standard to generate evidence to change clinical practice comes from randomized controlled trials (RCTs). Such models start from a tumor’s location in the body or histopathology rather than its underlying molecular makeup. Consequently, the generation of clinical evidence of predictive biomarkers or treatments targeting specific subgroups becomes a more daunting task. In addition, testing targeted therapies in clinical trials is challenging in view of the establishment of statistical significant effects, or recruitment of sufficient numbers of patients (8). Statistical significance may still occur in case the treatment has a large effect size and the incidence of the targeted group is sufficiently high in the total treatment population, or in case trials are designed to include a larger number of trial participants. However, the latter would increase costs, at times when drug developers are looking for savings. The decline in healthcare budgets coupled with escalating R&D costs and complexities has convinced stakeholders that the traditional models for drug development applied to precision oncology are unsustainable and may no longer be suitable to tackle coming challenges (9, 10).

The life science industry witnesses a history of huge challenges, whereby stakeholders adapted or evolved accordingly. For instance, the everlasting call for more effective therapeutics along the pharmaceutical crisis led to the emergence of alternative models for working together (11, 12). Likewise, the current complexities brought about by data-intensive precision oncology research outweigh the efforts possible within the walls of single organizations. The generation of clinical evidence in genomic diverse and geographically dispersed groups of patients requires access and linkage of massive amounts of data, including various types of “-omics” data extracted from biological samples, combined with lifestyle and clinical information, but also long-term side effects and survivorship issues, often referred to as “Big Data” (13). Yet, these are stored in distinct formats, originate from varying data sources, and are held by different stakeholders, complicating their integration. Present-day, data and samples generated from RCTs are not maximally leveraged by the cancer research community to achieve advances in precision oncology (14–18).

By pooling data from completed studies, researchers have access to large cohorts of patients, providing more statistical power to draw meaningful conclusions for patients. For example, data can be mined to allow *post hoc* subgroup analysis and thereby increase the precision of estimates of treatment efficacy, validate gene signatures, detect safety problems undetectable in smaller populations, generate new biological insights and increase the efficiency of R&D for instance, both in terms of time and costs, by avoiding duplicating trials and coming to better trial designs (19, 20). Volume enables greater understanding of the complexity of tumors, and the same holds true for samples: to create a comprehensive catalog of genes that acquire driver mutations in 2% or more of patients with cancer, Lawrence et al. suggests that more than 100,000 cancer samples need to be analyzed (21). Consequently, besides health information technology advances, it is critical to engage all stakeholders and share data and samples across research institutes to harness the potential of vast quantities of patient data that are currently locked away. It is against this backdrop that several groups and organizations have initiated collaborations to innovate the clinical research paradigm in oncology research.

With human samples being estimated worth more than diamonds, and data being handled as a new type of currency, appropriately managing these valuable patient resources is of utmost importance (22). In this paper, we theoretically describe different strategies for increased sharing of patient data and material that have been installed over the past decade. In parallel, a number of examples of these models are described. We zoom in on an emerging type of collaborative data sharing models in precision oncology that aims to combine omics and clinical data to address the current clinical research challenges: omics screening platforms. Finally, we introduce a tiered model to share patient data and samples, with appropriate consideration for patient and commercial confidentiality.

## Materials and Methods

This study is based on a scoping literature review. A search in the PubMed database using a combination of medical subject headings and text-words was performed from September 2016 to March 2017. The following key words and synonyms were used: data sharing, big data, biobanks, clinical research, clinical trial, precision oncology, and precision medicine. After removing duplicates, the remaining papers were screened in a stepwise manner based on title, abstract, and full texts. Included were papers where the content was clearly linked to the key words. Excluded were non-English papers. Key publications were selected in agreement with experts. Further, the reference list of the articles was checked to include additional articles. Besides examples from the literature, additional examples were included upon recommendation of experts being academics involved in clinical oncology research [e.g., omics screenings platforms and the Aide et Recherche en Cancérologie Diggestive (ARCAD) database]. Additionally, selected initiatives were discussed in a semi-structured way with multiple experts (oncologist, academics, and industry representatives) and websites of official organizations were screened to acquire in-depth knowledge. Not all models are specifically geared to clinical (oncology) research data, for instance general models for genomic data sharing [e.g., European Genome-phenome Archive (EGA) or database of Genotypes and Phenotypes (dbGaP)]. For cancer, however, being a genetically driven disease, genomic data sharing is of high importance to unravel the genomics underlying the disease, illustrated by the fact that these models are frequently being deployed in this context. Therefore, models that are—or could potentially be—of relevance for precision oncology research were also included.

## Results

In total, 374 articles were found through the search strategy. After applying the inclusion and exclusion criteria 38 articles relating to data sharing were withheld. Another 50 articles, reports and/or websites from institutions complemented these, which were recommended by experts or found through the reference method. Of these, 19 key articles provided insights on DSMs (Table 1). Similar to the studies by Wilhelm et al., Sydes et al., and Green et al., we classify two main strategies: “open access” models characterized by absence of a decision maker, and “controlled access” models (17, 23, 24). Where the former enables scientific peers to replicate or conduct new research without barriers, the latter imposes some form of control, as we will see sometimes for good reasons. Using this framework, examples were grouped in appropriate categories depending on their access strategy (i.e., open versus closed), deciding body (i.e., donor, provider, independent body, provider, and requester), and if possible location of database or biobank (centralized or federated). Our proposed sub-classification builds further on the four models proposed by Mello et al. combined with the other literature (25).

**Table 1:** Examples of different data sharing models with respective benefits and drawbacks.

Model	Advantage	Disadvantage	Reference
	**Open access strategy**		
Open access	No selective access, enables research without barriers; data sharing at relatively low costs and little administrative burden	No benefit-risk balancing; magnified risks in terms of misuse of data (no assurance that sound scientific methods are used); requires tools and resources for freely downloadable large, heterogeneous and complex datasets; no direct contact between data provider and requester impeding to provide information on the dataset; less suitable for datasets with high privacy risks	(17, 23, 25–28)
	**Controlled access strategy**		
Provider	Pre-specified set of criteria should ensure a transparent system; possibility to appeal to an independent board	Lack of full transparency or assurance of impartiality; difficult to identify data holders	(25)
Catalog	Clear overview of types of data held by different study teams; allows data generators to maintain autonomy	Datasets obtained on different consent forms complicated reuse	(29–31)
Partnership	Conduct of research in accordance with requirements of both parties; benefit-sharing strategies	Complex negotiations; increased timelines before project start	(11, 14, 32, 33)
Gatekeeper	Data provider cannot veto a request; transparent procedure; full assessment of scientific request and requester; apply benefit-risk balance test data sharing and share minimum data necessary for the request; communication portal between data provider and data requester	Costly (infrastructure, administration, maintenance; curation costs; human resources; opportunity costs); potentially time-consuming procedure	(23, 25, 26, 34–36)
Database query	No direct data sharing, thus can be applied for (personal or commercially) sensitive data; analyses are conducted by original study team who are most familiar with the nuances of the dataset; not limited by particular formats	Little control and transparency on executed queries; resource-intensive for data holders; potentially considerable wait times for requesters.	(25, 27, 30)
Donor controlled	Patient engagement and empowerment; effective reuse of data with explicit consent of the donor	Additional burden (increased resources for health literacy; infrastructures to manage patient preferences…)	(37, 38)

**Figure 1:**
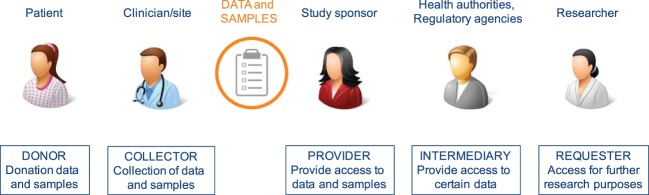
Chain of stakeholders involved in the process of sharing of clinical patient data and samples.

**Figure 2:**
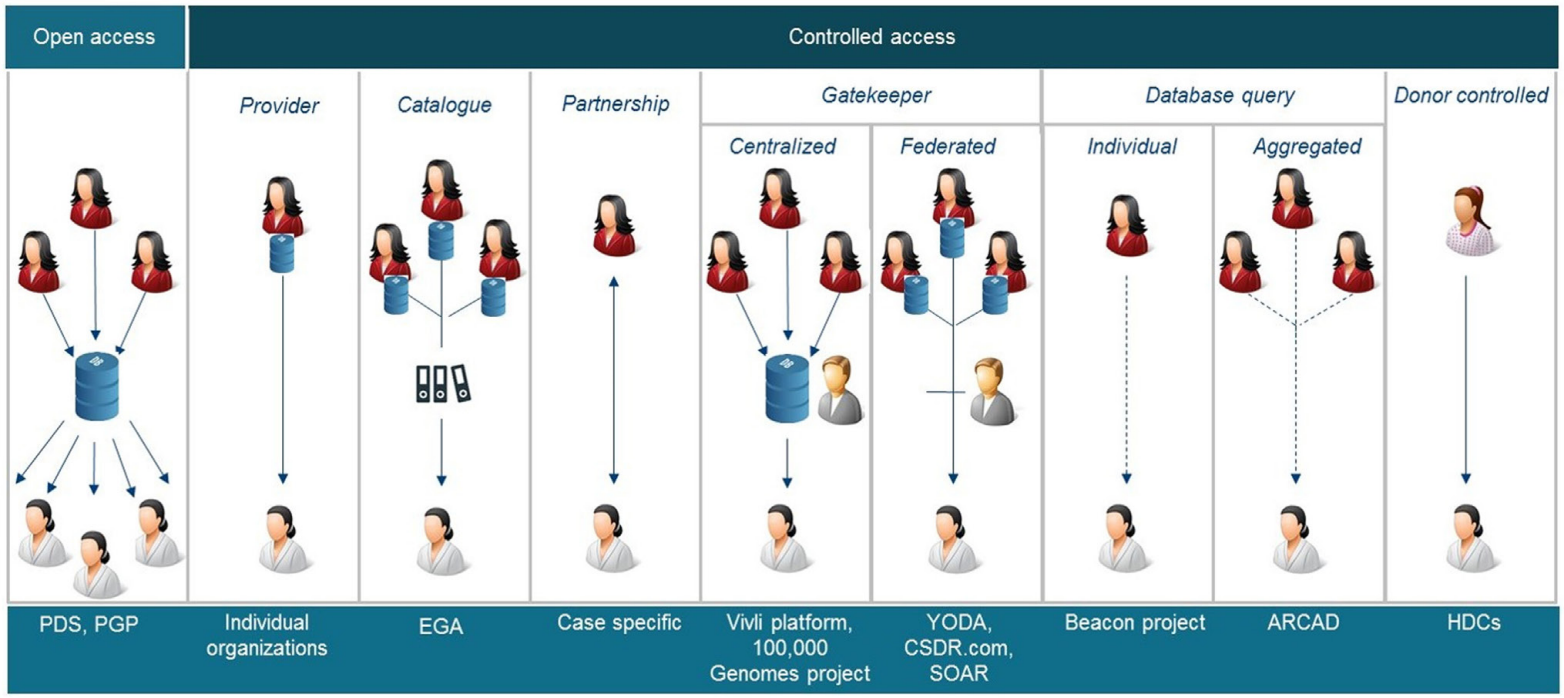
Schematic overviews of nine different data sharing models identified. PDS, project data sphere, PGP, Personal Genome Project, EGA European Genome-phenome Archive, YODA Yale University Open Data Access, CSDR.com ClinicalStudyDataRequest.com, SOAR, Supporting Open Access Research, ARCAD Aide et Recherche en Cancérologie Diggestive, HDC health data cooperatives. Some of these models are also applicable to share samples.

All stakeholders involved in clinical research have different roles/responsibilities in the process of data and sample sharing toward the common goal of improving patient benefits. In general, the sharing process (Figure 1) can be defined in a number of iterative steps; donors providing data or samples to the collector; the collector providing the samples and/or data to the sponsor, who stores them in a database and/or biobank; data providers (sponsors of clinical study or database or biobank); data provider making data or samples upfront available, or requesters finding the data or material, requesting access *via* intermediary or directly to provider, negotiating, and—upon agreement—receiving the requested data or material by the requester. Figure 2 shows a schematic overview of nine types of DSMs, representing numerous data sharing initiatives as identified in literature, which aim to facilitate the sharing process for clinical research data. Some of these models are also used in practice to provide access to patient material. Table 1 provides an overview of all discussed models with the respective benefits and drawbacks.

These models provide varying levels of control, access to data and/or samples (Table 2), and different types of relationship between the donor, the data provider, i.e., the primary study team or sponsor, and the data requester, i.e., the researcher of the secondary project (Figure 1). Different types of data can contain or occur in all levels of identifiability; however, it is generally accepted that human material and genomic information consider identifiable data since they entail all of one’s individual characteristics, and in addition, also personal information of relatives. Whether genetic and omics (genomes, transcriptomes, proteomes, exomes, epigenomes, and other types of similar information) data are classified as “primary” or “inferred” data depends on the level of investment the researchers made to generate, analyze, and report the data.

**Table 2:** Categorization of data and material according to (A) level of identifiability or encryption to safeguard the protection of an individuals’ identity, and (B) nature of the data and material.

	Category	Explanation
A	Identifiable data	Data that can be attributed to a specific data subject without the use of additional information
	Coded/pseudonymized data	Data processed in such a manner that the personal data can no longer be attributed to a specific data subject without the use of additional information
	De-identified/anonymized data	Data that cannot be attributed to a specific data subject

B	Material	Blood, saliva, tumor tissue…
	Primary patient data	The raw data underlying the results that enable reproducing the research
	Inferred, derived patient data	Data created by an (intellectual or financial) investment on the part of the primary research team
	Report of results	Summary of research data

### Open Access Models

Open access models are characterized by the absence of any review panel or decision maker. Researchers submit data which are available for download either directly or after a simple registration procedure. The fields of genomics have paved the way for fully open access databases, with the publicly funded Human Genome Project, characterized by the immediate and proactive publication of the human genome sequence, at the forefront (48). In general, only data accompanied with a consent for open sharing for research uses can be deposited in publicly available databases, such as the National Human Genome Research Institute and the European Bioinformatics Institute (EMBL-EBI) publicly funded Catalog of Published Genome-Wide Association Studies (GWAS Catalogue) (49), or the publicly funded Ensembl (50) database specifically tailored to store genomic, transcriptomic, proteomic, or sample data. In the Personal Genome Project (PGP), initiated more than a decade ago at the Harvard Medical School, genomic data from volunteers are openly shared, with the explicitly acknowledgment that it is impossible to guarantee privacy or anonymity. Therefore, the PGP appeals only to participants willing to waive any privacy expectations, through its so-called “open consent” (51). To further accomplish its goal of developing a publicly accessible dataset, the PGP makes use of creative commons licenses to share participants’ data and samples with minimal access restrictions (52).

Similar open access regimes are being deployed to share clinical trial data. The Project Data Sphere (PDS), a nonprofit initiative launched in 2014 and funded by the CEO Roundtable on Cancer, allows researchers to share, integrate, and analyze individual patient data (IPD) on a simple web-based platform (26). Many of these datasets can be downloaded onto researchers own computing environments, allowing much flexibility. To do so, users must register and accept a responsible use agreement. Besides data access, authorized users have access to SAS analytical tools to assist with data analysis and are provided access to templates of legal agreements. Data are submitted mostly after publication of trials to protect commercial interests, and to protect trial participants’ privacy, only after de-identification of any personal information. PDS proposes a de-identification strategy that satisfies legal requirements (the expert determination method of the HIPAA Privacy Rule is the preferred method (53)); however, final responsibility resides within the data provider (54). Additionally, other clever de-identification strategies for clinical trial data are proposed (55). By renouncing any form of control by an organization, the platform minimizes barriers to access and share data, and hopes to maximize potential benefits. Concerns have been expressed however, that unrestricted access to clinical trial data would lead to unskilled analysis and thus to flawed results. Such papers containing fallacious insights could be the basis of (pressured) misleading regulatory actions potentially harming patients (25, 27, 28). However, it is recognized that this model may be less suited for disclosing data of trials for rare disease or sensitive data where identification risks may be higher, i.e., genomic data from clinical trials (17). At present, the PDS contains data from almost 100,000 research participants from 116 trials provided by academia, government and industry sponsors. Just recently, PDS initiated alliances with Merck KGaA to jointly lead the Global Oncology Big Data Alliance (GOBDA) (56). GOBDA will enrich PDS by including data from rare tumor trials, experimental arm data and real-world patient data and leverages its potential by application of big data analytics.

A similar open access model has been introduced by the EU regulator in its flagship policy 0070. Here, the European Medicines Agency (EMA) commits to proactively publish clinical reports of all initial marketing authorization applications submitted after 1^st^ of January 2015 on the publicly available website https://clinicaldata.ema.europa.eu/web/cdp/home (57). Besides this user-friendly tool to get access to clinical reports, their use is further governed by two different terms of use (ToU) attestations. The applicable ToU depends on the intended use and information contained in the reports, which can be for on-screen view only when it considers general information purposes, or for a full download for academic and non-commercial research purposes (58). In order not to interfere with the Agencies’ decision-making process, documents will be published once the decision about a market authorization is made. Further, to anonymize published data from the clinical reports, personal data are redacted. Also, companies’ commercially confidential information (CCI) can be redacted, although in general, the Agency does not consider clinical data (i.e., clinical reports and IPD) as CCI. The EMA is committed to also share (whenever possible anonymized or otherwise pseudonymized) IPD in a later phase via this website.

To the best of the author’s knowledge, no open access regimes for clinical samples were found. One of the potential reasons might be captured in the following quote from a biostatistician in an academic research organization: *“If you talk about sharing samples, this is an action that cannot be repeated forever considering their perishable nature. You need to have more governance on deciding what the best purpose and the best timing is to re-use samples.”*

### Controlled Access Models

Besides a pure open access model, a more restrictive approach is applied in the controlled access models. Here, some form of control is exercised by either the donor (i.e., patient), the data provider (i.e., initial organization), or an independent party. This control allows for balancing the benefits and the risks of the data sharing: does the value gained from providing the data and executing the research outweigh the risks in terms of potential privacy breaches or competitive concerns? Six different controlled access models can be differentiated.

#### The Data or Sample Provider in the Driver Seat

While advances in precision oncology research depend among other things on the appropriate integration and retrospective analysis of patient data, it also often depends on the willingness of the providers (i.e., the custodians) to share “their” data or samples. Although it is generally accepted that sponsors or research teams can from an intellectual property (IP) point of view not *own* these resources, whoever possesses the data or samples physically, *controls* them and may determine whether and by whom its benefits can be tapped.

Under the traditional regime, third parties’ access to and use of clinical trial data is subject to the original trial sponsors’ authorization and can be granted to individual datasets on a case-by-case basis, mainly according to some formal mechanism laid down in the organizations’ policy. There is only little transparency, however impartiality (i.e., avoidance of selective access) is guaranteed as far as possible by having a mechanism for approval that is bound by a set of clearly defined criteria. In addition, the conditions for access in case of a positive decision should be declared in advance. In case of a negative decision, the rationale should be documented and publicized, which may in some organizations be appealed to a Data Access Committee (DAC) that takes a final decision. As such, the model aims to prevent data providers to impede data sharing for non-legitimate reasons.

For a long time, sharing of patient-level clinical trial data happened too often through informal processes, with the study sponsor in control of the decision of whether to share or not. The molecular disease classification of colorectal cancers is a case in point. When the first anti-epidermal growth factor receptor (EGFR) antibody therapies for colorectal cancers were brought to the market by industry, there were no subpopulations identified (59). It was only shortly after, through re-analysis of the industry-driven trials by academic investigators, that the association was made between activating mutations in the K-RAS gene and a lack of response to anti-EFGR inhibitors. This lead to a subdivision in responders (wild-type K-RAS) and non-responders (K-RAS mutations), and ultimately to a repurposing of the drug restricted to the responders accounting for approximately 60% of the total previous population (60). Later, other academics bundled forces to investigate the effects of other downstream mutations (PIK3CA, B-RAF, and N-RAS) on the efficacy of an EGFR inhibitor, cetuximab (erbitux, Merck KGaA), and, once again, confirmed low response rates demonstrating these to be negative predictive biomarkers (61). Today, taking all subpopulations together, the number of patients not benefiting from treatment was increased to almost 60% of the initial population, 60% that could otherwise be exposed to serious side effects when treated with anti-EFGR therapies. However it took more than 3 years before these new findings were picked up by the industry, to re-analyze the original trial data, and to confirm the result (62). This illustrates that in silo approaches, insufficient data sharing, and poor academia-industry interactions result in sub-optimal or delayed introduction of the latest scientific results into clinical practice.

The same seems to be true when it comes to clinical trial samples, as explained by an academic researcher often involved in clinical trials with oncologists and pharmaceutical companies: *“oncologists think, even from 20 years ago, ‘these samples were collected by me 20 years ago, I can decide who can do what with them’, and it is the same what the company will say: ‘I collected this, I paid for this, so I can decide who accesses it’, (*…*) I think that after a certain amount of time, you should take (this decision) away from these parties.”*

#### Catalogue

Catalogues, for instance public databases like the EU EudraCT database or the US ClinicalTrials.gov, containing metadata on organizations’ individual datasets, can help to identify the holders of clinical trial data and samples as a starting point for the access approval process. However, control still resides with the initial provider. Information found in catalogs is often only limited and the functionalities of the navigation interfaces can be improved, as said by a biostatistician from an academic research organization: *“The current trial registration tools are insufficient; individuals have a hard time to extract the correct information from the data as they are being entered now.”*

Besides these non-detailed databases, also metadata of more detailed datasets, including genomic or genetic datasets, can be found on public websites. Such data may be distributed across databases and computers around the world, virtually connected through software interfaces that allow seamless, controlled access. The EGA, launched in 2008 by the EMBL-EBI, goes further than merely cataloging data by also archiving and brokering data from data submitting organizations (29). The EGA provides an overview of studies for which participants have consented to their data being shared for research uses—but not for full, open public release. Access to individual-level biomolecular and phenotypic data can be requested, after which the data access decisions are made by the DACs of the submitting institution, not by the EGA (29). Consequently, the model allows data submitting institutions to maintain autonomy. The International Cancer Genome Consortium for instance, launched in 2008 to generate comprehensive catalogs of genomic abnormalities, uses the EGA to make its data available to the entire research community as rapidly as possible under particular access conditions (63). The EGA has similarities with its US variant, the dbGaP provided by NCBI (64). However, the dbGaP does not work with a de-centralized access-granting system since access decision are made by the National Institutes of Health.

When it comes to samples, biobank networks like the publicly funded pan-European BBMRI-ERIC initiative, aim to improve the accessibility and interoperability of existing sample collections (65). After registration on a public website, a web-based query tool provides an overview on available samples and associated medical data in the BBMRI catalog. Submitted research requests undergo ethical and scientific review by the BBMRI-ERIC Ethical and Scientific Review Board, respectively, after which the final access decision is made by the local biobank’s access committee.

#### Partnership

When a research project with a request for data is of sufficient scientific value for the data provider (e.g., the sponsor), he may decide to enter into collaboration with the requester rather than merely providing the data, and the same is true when it comes to sharing clinical samples. The Vice President Global medical affairs of a large pharmaceutical company explains: *“If an external researcher or co-operative group has an idea involving retained samples and they submit it to the company, we could potentially enter into a collaboration.”*

When initiating a collaborative project with existing data or samples (i.e., “retrospective model”), both parties must come to mutual agreements on the use of and access rights to pre-existing and newly generated data, publication of research results, and—sometimes the most complex—on pre-existing and resulting IP. However, the associated iterative negotiation processes are time consuming, resource, and labor intensive. To aid these discussions, partnership toolkits and standardized agreements have been developed (66). Still, the lack of formal mechanisms to make partners work together is regretted by a general manager oncology from a large pharmaceutical company: “*I hope that, by some (intervention) from the authorities, these discussions or negotiations could be taken more under an umbrella, making it easier for everybody, because now many researchers and companies don’t understand this anymore; it starts to be a legal department at the hospital and a legal department here, and there is no science involved anymore*.*”* Collaborations span a range of models and can occur in the form of interdisciplinary academic initiatives, academia-industry (11, 67), industry–industry, or more complex multi-stakeholder partnerships (5, 68). Specific collaborations with a high public interest [e.g., biomarker research in oncology (69)] could be incentivized through financial, legal, or organizational support, or in the form of private–private partnerships (PPPs) which have their own IP and data sharing specifications (70).

The retrospective nature of most conventional data sharing models limits the data to be used rather exploratory or for hypothesis generating research. In another approach, partners seek each other and establish a new database/biobank with the aim to be widely accessible for multiple research purposes (i.e., “prospective model”). Especially in precision oncology, a number of collaborative initiatives has been set up to develop in a prospective fashion sustainable, high-quality, and integrated patient data collections, leveraging linked clinical and -omics data to accelerate research, facilitate patient-centered clinical trials and/or provide clinical insights that can be fed back to patients. A member of an independent review board (IRB) from a renowned, large data sharing model stated the following in this respect: “*We are seeing more of ‘pre-competitive collaborative research’ because the blockbuster days are gone and everybody needs the same basic data so why not just work together in a public-private consortium to move everything forward and when you get enough data and samples then you can go back to your competition and see who gets the product out first.”*

Performing clinical trials in smaller treatment populations increasingly pivots around operational challenges, namely how to perform large-scale sample characterization for patient screening. Collaborative platforms propose to jointly organize such screening in a precompetitive setting, for instance in Europe the European Organisation for Research and Treatment of Cancer (EORTC) Screening Patients for Efficient Clinical Trial Access initiative (32) or the US National Cancer Institute-Molecular Analysis for Therapy Choice (NCI-MATCH), or the for-profit, multi-institutional oncology research information exchange network (42) (Table 3). The opinion of a medical doctor illustrates this: *“A consequence of precision medicine is that pharmaceutical companies will need to compromise; they might need to enter into collaboration agreements with these types of screening platforms because it will be too difficult to have access to certain patients, otherwise their business is finished.”*

**Table 3:** Non-exhaustive overview of prospective, collaborative -omics screening platforms to facilitate clinical research in precision oncology.

Platform	Organization(s)	Location	Omics analysis	Tumor	Reference
AURORA	BIG	Belgium	NGS for a panel of 411 cancer-related genes	Breast	(39, 40)
Exactis	PMT	Canada	No information publicly available	Breast, lung, colorectal, ovarian, melanoma, prostate	(41)
ORIEN	Moffitt Cancer Center, The Ohio State University Comprehensive Cancer Center, Arthur G. James Cancer Hospital, Richard J. Solove Research Institute in Columbus	US	No information publicly available	All malignancies	(42)
NCI-MATCH	NCI	US	NGS	Solid tumors	(43)
PMT initiative	Exactis Innovation	Canada	-omics platforms	Colorectal, lung, melanoma, breast	(41)
SPECTA	EORTC	Europe	-omics platforms	Colorectal, lung, brain, melanoma, rare, prostate	(32)
Stratified Medicine Platform 2	Cancer Research UK	The UK	No information publicly available	NCSLC	(44)
The CPCT	Nederlands Kanker Instituut-Antoni van Leeuwenhoek Ziekenhuis, Erasmus MC Kanker Instituut, UMC Utrecht	The Netherlands	HiSeq Xten Illumina (WGS) (45)	All malignancies	(46)
U-can	Uppsala University	Sweden	WGS, SNP analyses, RNA Seq	Colorectal, leukemia, lymphoma, prostate, brain, gynecological, neuroendocrine, breast	(47)

*CGH, comparative genomic hybridization; EORTC, European Organisation for Research and Treatment of Cancer; NCI-MATCH, National Cancer Institute-Molecular Analysis for Therapy Choice; NGS, next generation sequencing; NCSLC, non-small cell lung cancer; ORIEN, oncology research information exchange network; PMT, personalize my treatment; RNA, ribonucleic acid, SNP, single nucleotide polymorphism, SPECTA, screening patients for efficient clinical trial access; The CPCT, the center for personalized cancer treatment; WGS, whole genome sequencing*.

#### Gatekeeper Model

Under this regime, access to data is not at the data providers’ discretion but may be granted by a distinct entity. Often, an IRB acts as a neutral intermediary that decides on the access to specific data sets. It does so, based on the scientific soundness of the research proposal submitted by researchers, on the expertise of the team and taken into account to benefit-risk balance of providing the data for that specific purpose. In this model, a central entity can act as a repository to collect and house existing clinical trial data (“centralized model”), or as a web-based search system providing general information about available data sets, however the data themselves are stored by the data providers (“federated model”). Such approaches support procedural transparency since they obligate to motivate decisions for non-disclosure. Industry representatives on their side, favor this approach compared to an open access model, because it allows initiating a dialog with requesters to explain questions on datasets, certain findings, or rationales for trial adaptations.

The industry’s commitment to data sharing builds on the gatekeeper model (71) and is implemented by single organizations (i.e., the public–private funded Yale University Open Access (YODA) project of Johnson & Johnson (72), or the publicly funded Supporting Open access Research (SOAR) initiative (73)) and by collaborative platforms such as the ClinicalStudyDataRequest.com platform (74). These platforms provide access to data through a password-protected secure internet connection; however, data are not downloadable. Costs of the platform are born by the data providers, according to Rockhold F. et al *“An investment of about $30,000 to $50,000 per year is needed for an academic sponsor to list up to 20 studies on the request site and for up to 10 research projects to be undertaken using data in the secure access site,”* consequently “*The overall costs can seem disproportionately high for sponsors or investigators with few trials*.,” deferring other organizations from joining the platform (34).

The Vivli platform, sponsored by the Multi-Regional Clinical Trials Center of Brigham and Women’s Hospital and Harvard University (MRCT Center), aims to create a singly portal, merging the myriad of existing platforms of sponsors enabling analysis of multiple datasets (35). Vivli is flexible for data providers since its secure computing environment enables aggregation of both centrally as well as federally hosted dataset. The platform will curate data from existing platforms into structured, computable metadata to allow for more accurate searches. On top of clinical trial data, ViVli aims to develop over time, the capacity to also share other data such as real-world data and omics data (35). Data shared through such secure platforms is free of charge, however, some have voiced concerns about the costs and resources required to secure and sustain this model (34). A drawback is that these platforms often do not allow access to individual genomic data or samples.

The gatekeeper model is advised by international recommendations for biobanks to share human material (75). Consequently, many local biobanks operate with an appointed IRB. However, it is seen that access arrangements of many biobanks lack completeness, not at least when it comes to the establishment of independent access mechanisms to maximize the value of clinical sample collections (76).

Some initiatives, like the (public and privately funded) 100,000 Genomes Project, are focused on enabling access to genomic data linked with continually updated clinical data of cancer patients (77). External scientists must apply for membership of the 100,000 Genomes Project research community. Upon approval of a research project by an IRB (so-called “Access Review Committee’, ARC) and an internal Ethics and a DAC, members can access the data for free on the project’s secure servers, pharmaceutical companies on their part have to pay a substantial fee (78). This project is set up by Genomics England, a company owned by the Department of Health. Both the whole genome sequencing data, clinical data and any IP generated during the project are owned by Genomics England, who proclaims to license this to third parties under favorable terms. Any profits made ought to be reinvested into genomics medicine (78).

#### Database Query

In an alternative, more restrictive model, data are not shared directly and custody is retained, rather the research questions or a copy of an analytical computer program is sent to the data provider, who runs the query and sends back the computed results to the requester. This so-called “database query” model is believed to be more secure since fewer copies of data—that can be attacked or stolen—are made (30). This model is useful to access sensitive data (e.g., for genome analysis) by requesting results from queries on personal identifiable data, since the latter fall out when the analytical results are presented to the requester. Datasets can be queried individually, or at the aggregate level. A possible limitation of the model may include its lack of transparency, precluding requesters from verifying that the results they receive are valid.

In 2012, a group of gastrointestinal oncologists bundled forces to launch the ARCAD Advanced Colorectal Cancer Database Project (79). This project, supported by public and private grants from industry, aims to bring together in one single database de-identified IPD from most of the recent prospective clinical trials in advanced colorectal cancer (“aggregated model”), including both industry and academic trials across all lines of therapy. Currently, IPD from almost 40 randomized trials comprising >35,000 patients are incorporated into the database. Data include baseline demographics, clinical and laboratory assessments (including relevant biomarkers), treatments, tumor measurements over time, and outcomes. Both ARCAD and non-ARCAD members are invited to propose further studies with a view to collaborative projects; however, the database will be analyzed by ARCAD statisticians and trialists (80). Research proposals will be examined by ARCAD review committee, and to respect the interest of data providers, all data providers are consulted before every analysis and have the freedom to withhold their trial data from any analysis.

BBMRI-ERIC suggest the use of the database query model in case industrial users want to access samples. Human samples can legally and ethically not be sold; however, industrial users may access and use specimens for the R&D of commercial products (65). BBMRI’s so-called “Expert Centers” are not-for-profit intermediate infrastructures set up as PPPs that will perform analysis of human samples at the request of industry, and subsequently make the data available that may be used in product development. The same model is suggested to be of use in a situation where researchers from different countries want to collaborate, but when country-specific legal restrictions on export of human material complicate international research. In such situation, expert centers act as “highways” for transnational research collaborations, meaning that samples will be analyzed in the country of origin, and only research data are shared (65).

The Beacon Project of the (public and private funded) Global Alliance for Genomics and Health (GA4GH) and ELIXIR, the on EU grants based infrastructure for life science data, is a more technology-savvy example of this model (81). The project aims to improve the discoverability of genomic data by making use of “beacons.” Beacons are online web services, tiny search functions added to databases, which allow users to query institutions’ databases to get specific allele-presence information. For instance, it allows questions in the form of “Do you have any genome with a ‘nucleotide x’ at position ‘y’ on chromosome ‘z’?” to which the beacon responds with either “yes” or “no.” The result of this query efficiently informs the user as to whether the variant of interest exists, and thus whether an access request for more detailed data would be deemed useful. As such, beacons are a first step toward greater openness and data sharing. By its federated approach—one single space allows querying across beacons set up by the member organizations—data providers maintain control.

#### Donor Controlled

In line with the European Commissions PerMed consortium recommendations and the revised EU data protection framework, both underlining the importance to enhance patients’ control over their own data (82), trial participants are advocating for more control over their own medical data (83). In this respect, privacy-enhancing techniques such as e-consent have been proposed to allow for a more dynamic interface where trial participants can manage their own data sharing preferences (84).

Health data cooperatives try to circumvent the inaccessibility resulting from data silos, by prospectively creating a trusted entity where individuals can safely store, control, manage, and share their own data. In this hypothetical model, participants themselves can thus decide to open up their data, and to whom they disclose it (37). In support of genomics research, similar programs have been proposed where individuals’ can donate their DNA and health records, analogous to organ-donor systems (38).

The growing interest of public and patient engagement in research is also reflected in the establishment of a number of patient-led biobanks, for instance the German Patients’ Tumor Bank of Hope (PATH) (85) which collects blood and tumor tissue with associated data from breast cancer patients over time. Decision to grant requesters access to the samples and data are made by its board which consists out of three breast cancer survivors (86). Such models, where donors control access, are considered ethically correct by a legal advisor from a clinical research center: *“Samples belong to the patient, and organizations get access to them through a study or a trial, (*…*) but they remain the property of the patient; the person who can decide what happens with the samples should be the person from whom the sample was collected*.*”*

### Toward a Sustainable Biomedical Sharing Ecosystem

The recent revolution in science and technology applied to medical research has left in its wake a trial of biomedical data and human samples; however, the opportunities remain largely unfulfilled. To harness these opportunities biomedical research organizations’ and pharmaceutical companies’ collaboration and innovation models should appropriately adapt. Not surprisingly, the debate is largely focused on the precision oncology research arena considering its high potential to leverage “Big data” and samples into meaningful solutions for patients.

Sharing such data is critical to scientific and medical progress, but is has been hampered because of legal, ethical, financial, strategic, and technical barriers. Fulfilling the legal/ethical requirements to protect participants’ privacy, or organizations’ confidentiality while guaranteeing incentives for investment in research, seems to conflict with an approach of openly sharing personal data and human material to advance scientific knowledge and achieve patient benefits.

From a policy perspective, the question is whether patients and society are better off under a regime that favors open data sharing over a regime of more controlled or very restricted data sharing. Further, should the sharing of clinical trial data and samples (openly or controlled) be mandated, and if so, how and to what extend should this be organized in a legal, ethical, and innovation-friendly way? To resolve this dilemma, a better understanding of different sharing models and their characteristics was deemed useful. Based on pre-existing literature and practical examples as well as expert opinions, we conceptualized a number of models.

While the primary goal of all models is to enable further research, it seems obvious that the open access approach mirrors this goal perfectly. Examples demonstrate that the open access model has been proven feasible, traditionally in the field of genomic research but now also for clinical trial data. Sharing genomic data from clinical research participants through this model remains more difficult, and this might be due to differences in applicable consent restrictions between non-clinical versus clinical trial genomic research projects. The impressive amount of trials submitted in PDS demonstrates that providers are willing to submit their data, underlining the success of the model. While guaranteeing the protection of privacy might be impossible when sharing genomic information, the PDS provides guidance about methodologies that can be applied to de-identify clinical trial data in compliance with legally prescribed standards (53).

Having appropriate safekeeping mechanisms in place to control sharing, by providing access only after fulfillment of certain conditions, for instance for privacy-sensitive data or data restricted by IP protection, remains a good alternative for keeping both patients’ and organizations’ interests safe. The traditional controlled access models have led to an emergence of numerous data and sample silos, undoubtedly at the expense of scientific advances. Comprehensive catalogs with different types of data and samples would provide a useful tool to identify the custodians of data and samples collections and determine whether access is of interest. However, current legally mandated trial data catalogs seem insufficient, especially to track down biomarker data or samples. Voluntary catalogs such as the BBMRI-ERIC model are to be applauded but it remains unclear to what extent this biobank catalog will contain information on sample collections held under the auspices of for instance for-profit trial sponsors, complicating the access to these valuable resources.

Partnerships remain a vital strategy in biomedical (oncology) research to maximize the value of resources that would otherwise remain untouched. Despite the willingness to collaborate, both academia as industry representatives indicated to regret the lack of systematic and coordinated approaches to enter into partnerships. Still too often, research projects are initiated based on personal contacts. The scale and opportunities brought forward by Big data, together with the complexities afforded by the level of precision we are aiming for in oncology, may be an inflection point: the need to study rare variants, the combinatorial complexity of treatments and the increasing number of stratified trials have led to the setup of prospective and precompetitive -omics screening platforms. It may not be practical in the future to work without collaborating with such models in order to conduct patient-centric trials to validate certain precision oncology treatments. For sure, the setup and maintenance of these platforms have their own difficulties, not at least high costs and resources to recruit and characterize a critical mass of patients, which is in turn essential to attract downstream research projects of which the revenues could again be invested. Another complexity centers on the quality of information. Where retrospective data from federated models can be informative, it can be questioned whether these data will meet regulatory standards to support and change clinical decision-making. This does not mean that such data should not be used; rather the results should be interpreted cautiously. Alternatively, to generate sufficient large collections of regulatory-grade patient data in a prospective fashion demands logistical solutions for instance for biobanking; however, at costs that might make it unaffordable.

An emerging trend to share clinical trial data is the use of the gatekeeper model. One of the reasons for this might be that it secures neutrality on the decision, and at the same time ensures some form of interaction between data generator and data requester. Each study has its limitations, who are best known by the researchers involved in the project. Not knowing these might introduce important confounding in secondary analysis. For instance, a good understanding of the conditions under which the data and samples were collected, the complex datasets, and specific statistical tests in biomarker studies in oncology is essential to ensure appropriate analysis. Through this model, the primary research team can provide this necessary guidance, or can be invited to join the secondary study. The huge costs of this model, however, renders it less attractive. Since sharing clinical samples is impossible through an open access model considering their physical nature, further encouraging the use of gatekeeper models to share clinical samples is useful. The relevant IRBs will need to make some additional decision relating to for instance prioritization of scientific projects on the basis of evaluation criteria (76).

Lastly, also federated models in which queries are sent to the original data or sample providers, thereby not necessitating an act of sharing sensitive information, are being adopted. Depending on the level of detail that can be queried, such models can be considered more secure. Consequently, these models are especially useful to address data protection issues or concerns about IP and competitiveness. Several important precedents have been set here by the oncology research community such as the Beacon Project where uncovered genetic variants from one institution can be linked to similar variants of other databases, increasing evidence on their clinical relevance and utility.

**Figure 3:**
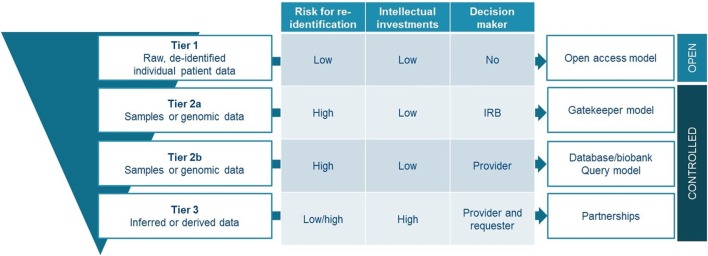
Proposed tiered-layered model to share and access clinical data and samples.

Data and sample sharing models have evolved over the past decades, now spanning a continuum from traditionally closed models up to full open access models. Different strategies will continue to exist and it is highly unlikely that completely open models will dominate future practices. However, in an era where data is driving future innovations, but the data sources are fragmented, finding appropriate models to share and collaborate on projects are quintessential. Several models are mapped here, describing various levels of control over the data, and different relationships between data providers and users. We believe that there is no universal or one-size-fits-all solution that should be mandated by policy makers. However, we would like to propose a tiered model for sharing that takes into account the characteristics attached to certain types of data and samples (Figure 3). The proposed model is tiered, as it offers a strategy depending on the legal, ethical, and strategic issues attached to the shared resources.

A first tier would be for everyone to share de-identified raw, IPD from clinical trials—indispensable information for verification of studies—by use of an open access model such as proposed by PDS. This approach requires in parallel solid processes for de-identification, exclusions to openly share datasets with high risk for re-identification, and the implementation of commonly agreed responsible use attestations. Additional tiers offer more detailed information made available upon request through controlled access mechanisms. For accessing samples or genomic information, it may be impossible to use an open access model considering their sensitive nature, unless patient would provide open consent which is highly questionable in the context of clinical research projects. We believe that gatekeeper models with independent oversight, would be most suited to organize data sharing for these types of patient resources (tier 2a). If for some reason, more control from the data and/or sample providing organization would be necessary; the database query model represents a good alternative (tier 2b). As a third tier, the setting up of partnerships should be promoted. These partnerships should aim to maximize the use of inferred or derived data, while addressing competitive concerns related to them. Promotion of partnership can be done for instance through the provision of structured contractual agreements of which a substantial part should be attributed to IP and benefit-sharing agreements. Similarly, both academia and industry engaging in precision oncology clinical research could benefit from such structured agreements for collaboration with an -omics screening platform.

Overarching all tiers, the further development of a standardized cancer ontology combined with catalogs or other search tools for metadata to make the providers of data or samples more findable is considered useful, in line with the first of four FAIR principles (87). Further, we support as a rule that all reporting of results based on research with shared data and samples should contain appropriate co-authorship, or at least attributions, to recognize and acknowledge the original data or samples holders (i.e., provider) (88). Finally, to increase donor’s control over their data and sample management, and to increase overall transparency—two key principles embedded in the upcoming EU General Data Protection Regulation (89)—the use of modern privacy-enhancing tools such as dynamic forms of e-consent should be further explored (83, 90).

Through the latter measure, patients would have an opportunity to become more actively engaged in the whole data sharing process. More generally, our proposed model aims to increase transparency and thus trust in the use and subsequent reuse of clinical trial data and samples, while maximizing benefits. As such, this model aims to respect patients who put themselves at risk by participating in a trial, and meets the obligation delineated in informant consents that the results from trials lead to the greatest possible benefits not necessarily for the participating patients but for future patients.

This study suffers from a number of limitations. First, the results are based on the author’s interpretation of the literature. Although in line with other articles, others might come to a different classification of the models. Second, we restricted our search to general data (and sometimes sample) sharing models and models specifically deployable in oncology, imposing a selection bias. Yet, other examples (fitting within this categorization) in other disease areas exist. Third, certain of these models relate to sharing genomic research datasets and not specifically to clinical oncology research data. Although useful, since the boundaries between both types of research are increasingly blurred in data-intensive precision oncology research, genomic data sharing has typically followed a liberal model, characterized by an open approach to freely share and exchange data (e.g., Bermuda Principles 1996).

Further unveiling the molecular architecture of cancer necessitates the inclusion of data resulting from multiple omic methods applied to patient samples. Hence, it is necessary to enlarge the current focus on clinical trial data sharing to include sharing of samples of which new information can still be extracted. Currently, efforts to encourage sample sharing are limited when compared to data sharing. To conclude, we propose a tiered-staged model for sharing of clinical trial data and samples that takes into account the legal, ethical, and strategic concerns. Such model can help spark the debate to come to commonly agreed solutions that aim to facilitate precision oncology research, an area that will maximally benefit from increased sharing. According to the Clinical Cancer Genome Task Team of the GA4GH: *“If we don’t concentrate our efforts (and dedicate substantial resources) to robustly improve data sharing, we risk undermining precision oncology’s capacity to deliver substantive advances for people with cancer.”* We believe our proposed model can increase these efforts and contributes to maximally achieve this aim. Organizations active in oncology drug development should think about an effective tiered-sharing strategy to maximize the value of the resources donated by patients, while not diminished the incentives to invest in research. Research shows that the drug development model has reached its innovation capacity, and this is especially true for precision oncology (7, 12, 91–93). The adopted open innovation practices by the research community—of which data sharing being one of the most pronounced ones—beholds the power to shift the current paradigm of siloed and fragmented clinical research toward scientific collaborations based on pooling of expertise, ideas and resources. Over time, this will contribute to a more efficient drug development model, advance science and aid in the fight against cancer.

## Author Contributions

SB wrote the original manuscript. All authors read, contributed, and approved the final manuscript.

## Conflict of Interest Statement

SB received a PhD scholarship of the EORTC. All other authors declare that the research was conducted in the absence of any commercial or financial relationships that could be construed as a potential conflict of interest.

## References

[1] Stewart B Wild C World Cancer Report 2014. Lyon: OMS – IARC (2014).

[2] DeVita VT Eggermont AMM Hellman S Kerr DJ. Clinical cancer research: the past, present and the future. Nat Rev Clin Oncol (2014) 11:663–9. 10.1038/nrclinonc.2014.153 25245981

[3] Khalil DN Smith EL Brentjens RJ Wolchok JD. The future of cancer treatment: immunomodulation, CARs and combination immunotherapy. Nat Rev Clin Oncol (2016) 13:273–90. 10.1038/nrclinonc.2016.25 26977780 PMC5551685

[4] Trusheim MR Berndt ER Douglas FL. Stratified medicine: strategic and economic implications of combining drugs and clinical biomarkers. Nat Rev Drug Discov (2007) 6:287–94. 10.1038/nrd2251 17380152

[5] Aitken M Global oncology trend report – a review of 2015 and outlook to 2020. IMS Inst Healthc Inform. Parsippany: IMS Institute for Healthcare Informatics (2016). 9 p. Available from: https://morningconsult.com/wp-content/uploads/2016/06/IMS-Institute-Global-Oncology-Report-05.31.16.pdf

[6] Masters GA Krilov L Bailey HH Brose MS Burstein H Diller LR Clinical cancer advances 2015: annual report on progress against cancer from the American society of clinical oncology. J Clin Oncol (2015) 33:786–809. 10.1200/JCO.2014.59.9746 25605863

[7] Hollingsworth SJ. Precision medicine in oncology drug development: a pharma perspective. Drug Discov Today (2015) 20:1455–63. 10.1016/j.drudis.2015.10.005 26482740

[8] Klauschen F Andreeff M Keilholz U Dietel M Stenzinger A The combinatorial complexity of cancer precision medicine. Oncoscience (2014) 1:504–9. 10.18632/oncoscience.66 PMC4278319 25594052

[9] Stupp R How Europe can develop better, cheaper cancer drugs. CancerWorld (2014):49–54.

[10] Said M Zerhouni E. The role of public-private partnerships in addressing the biomedical innovation challenge. Nat Rev Drug Discov (2014) 13:789–90. 10.1038/nrd4438 25359362

[11] Melese T Lin SM Chang JL Cohen NH Open innovation networks between academia and industry: an imperative for breakthrough therapies. Nat Med (2009) 15:502–7. 10.1038/nm0509-502 19424212

[12] Paul SM Mytelka DS Dunwiddie CT Persinger CC Munos BH Lindborg SR How to improve R&D productivity: the pharmaceutical industry’s grand challenge. Nat Rev Drug Discov (2010) 9:203–14. 10.1038/nrd3078 20168317

[13] Khozin S Kim G Pazdur R Regulatory watch: from big data to smart data: FDA’s INFORMED initiative. Nat Rev Drug Discov (2017) 16:306 10.1038/nrd.2017.26 28232724

[14] Hinestrosa MC Dickersin K Klein P Mayer M Noss K Slamon D Shaping the future of biomarker research in breast cancer to ensure clinical relevance. Nat Rev Cancer (2007) 7:309–15. 10.1038/nrc2113 17384585

[15] Chan AW Song F Vickers A Jefferson T Dickersin K Gøtzsche PC Increasing value and reducing waste: addressing inaccessible research. Lancet (2014) 383:257–66. 10.1016/S0140-6736(13)62296-5 24411650 PMC4533904

[16] The Clinical Cancer Genome Task Team of the Global Alliance for Genomics and Health. Sharing clinical and genomic data on cancer – the need for global solutions. N Engl J Med (2017) 376:2006–9. 10.1056/NEJMp1612254 28538124

[17] Green AK Reeder-Hayes KE Corty RW Basch E Milowsky MI Dusetzina SB The project data sphere initiative: accelerating cancer research by sharing data. Oncologist (2015) 20:464–e20. 10.1634/theoncologist.2014-0431 25876994 PMC4425388

[18] Hewitt RE. Biobanking: the foundation of personalized medicine. Curr Opin Oncol (2011) 23:112–9. 10.1097/CCO.0b013e32834161b8 21076300

[19] Lawler M Siu LL Rehm HL Chanock SJ Alterovitz G Burn J All the world’s a stage: facilitating discovery science and improved cancer care through the global alliance for genomics and health. Cancer Discov (2015) 5:1133–6. 10.1158/2159-8290.CD-15-0821 26526696

[20] Zhu C Santos C Ding K Sakurada A Cutz J Liu N Role of KRAS and EGFR as biomarkers of response to erlotinib in National Cancer Institute of Canada Clinical Trials Group Study BR. J Clin Oncol (2008) 21:4268–75. 10.1200/JCO.2007.14.8924 18626007

[21] Lawrence MS Stojanov P Mermel CH Robinson JT Garraway LA Golub TR Discovery and saturation analysis of cancer genes across 21 tumour types. Nature (2014) 505:495–501. 10.1038/nature12912 24390350 PMC4048962

[22] Pirnay JP Baudoux E Cornu O Delforge A Delloye C Guns J Access to human tissues for research and product development: from EU regulation to alarming legal developments in Belgium. EMBO Rep (2015) 16:557–62. 10.15252/embr.201540070 25851645 PMC4428037

[23] Wilhelm EE Oster E Shoulson I Approaches and costs for sharing clinical research data. JAMA (2014) 311:1201–2. 10.1001/jama.2014.850 24556937

[24] Sydes MR Johnson AL Meredith SK Rauchenberger M South A Parmar MK. Sharing data from clinical trials: the rationale for a controlled access approach. Trials (2015) 16:1–6. 10.1186/s13063-015-0604-6 25872927 PMC4369803

[25] Mello M Francer J Wilenzick M Teden P Bierer B Barnes M Preparing for responsible sharing of clinical trial data. N Engl J Med (2013) 369:1651–8. 10.1056/NEJMhle1309073 24144394

[26] Bertagnolli M Sartor O Chabner B Rothenberg M Khozin S Hugh-Jones C Advantages of a truly open-access data-sharing model. N Engl J Med (2017) 376:1178–81. 10.1056/NEJMsb1702054 28328337

[27] Spertus JA The double-edged sword of open access to research data. Circ Cardiovasc Qual Outcomes (2012) 5:143–4. 10.1161/CIRCOUTCOMES.112.965814 22438460

[28] Eichler H-G Abadie E Breckenridge A Leufkes H Rasi G Open clinical trial data for all? A view from regulators. PLoS Med (2012) 9:3–4. 10.1371/journal.pmed.1001202 PMC3323505 22505851

[29] Lappalainen I Almeida-King J Kumanduri V Senf A Spalding JD Ur-Rehman S The European Genome-phenome Archive of human data consented for biomedical research. Nat Genet (2015) 47:692–5. 10.1038/ng.3312 26111507 PMC5426533

[30] The Global Alliance for Genomics and Health. A federated ecosystem for sharing genomic, clinical data. Science (2016) 352:1278–80. 10.1126/science.aaf6162 27284183

[31] Savage N. Getting data sharing right to help fulfill the promise of cancer genomics. Cell (2017) 168:551–4. 10.1016/j.cell.2017.01.003 28187273

[32] Lacombe D Tejpar S Salgado R Cardoso F Golfinopoulos V Aust D European perspective for effective cancer drug development. Nat Rev Clin Oncol (2014) 11:492–8. 10.1038/nrclinonc.2014.98 24935010

[33] Ferri M Preparing for responsible sharing of clinical trial data. N Engl J Med (2014) 370:484–5. 10.1056/NEJMc1314515 24476448

[34] Rockhold F Nisen P Freeman A Data sharing at a crossroads. N Engl J Med (2016) 375:1115–7. 10.1056/NEJMp1608086 27653563

[35] Breier B LI R Barnes M Sim I A global, neutral platform for sharing trial data. N Engl J Med (2016) 374:2411–3. 10.1056/NEJMp1605348 27168194

[36] Krumholz H Waldstreicher J The Yale Open Data Access (YODA) Project – a mechanism for data sharing. N Engl J Med (2016) 375:403–5. 10.1056/NEJMp1607342 27518657

[37] Hafen E Kossmann D Brand A. Health data cooperatives – citizen empowerment. Methods Inf Med (2014) 53:82–6. 10.3414/ME13-02-0051 24514946

[38] Lu WJ Flockhart DA. Personal DNA donation to energize genomic medicine. Clin Pharmacol Ther (2014) 95:129–31. 10.1038/clpt.2013.131 24448457

[39] BIG AURORA. Aiming to Understand the Molecular Aberrations in Metastatic Breast Cancer: Where Do We Stand? (2016). Available from: http://www.bigagainstbreastcancer.org/news/aurora-aiming-understand-molecular-aberrations-metastatic-breast-cancer-where-do-we-stand/

[40] Zardavas D Maetens M Irrthum A Goulioti T Engelen K Fumagalli D The AURORA initiative for metastatic breast cancer. Br J Cancer (2014) 111:1–7. 10.1038/bjc.2014.341 25225904 PMC4229627

[41] Exactis. Personalize My Treatment (PMT Initiative). (2017). Available from: http://www.exactis.ca/pmt-en

[42] ORIEN. Oncology Research Information Exchange Network. (2016). Available from: http://oriencancer.org/#about

[43] National Cancer Institute. NCT02465060 Clinical Trial. (2017). Available from: https://www.cancer.gov/about-cancer/treatment/clinical-trials/search/view?cdrid=773118

[44] Cancer Research UK. Stratified Medicine Programme. (2016). Available from: http://www.cancerresearchuk.org/funding-for-researchers/how-we-deliver-research/our-research-partnerships/stratified-medicine-programme

[45] Stichting Hartwig Medical Foundation. (2017). Available from: http://www.hartwigmedicalfoundation.nl/en/

[46] The Center for Personalized Cancer Treatment – CPCT. (2016). Available from: http://www.cpct.nl/nl/over-cpct/

[47] U-CAN – Uppsala University. (2016). Available from: http://www.u-can.uu.se/?languageId=1

[48] Lander ES Linton LM Birren B Nusbaum C Zody MC Baldwin J Initial sequencing and analysis of the human genome. Nature (2001) 409:860–921. 10.1038/35057062 11237011

[49] MacArthur J Bowler E Cerezo M Gil L Hall P Hastings E The new NHGRI-EBI Catalog of published genome-wide association studies (GWAS Catalog). Nucleic Acids Res (2017) 45:D896–901. 10.1093/nar/gkw1133 27899670 PMC5210590

[50] Ensembl Genome Browser 90. (2017). Available from: https://www.ensembl.org/index.html

[51] Lunshof JE Bobe J Aach J Angrist M Thakuria JV Vorhaus DB Personal genomes in progress: from the human genome project to the personal genome project. Dialogues Clin Neurosci (2010) 12:47–60. 20373666 10.31887/DCNS.2010.12.1/jlunshof PMC3181947

[52] Personal Genome Project: PersonalGenomes.org *– Sharing Policies* (2017). Available from: http://www.personalgenomes.org/#project-guidelines

[53] Guidance Regarding Methods for De-identification of Protected Health Information in Accordance with the Health Insurance Portability and Accountability Act (HIPAA) Privacy Rule. (2017). Available from: https://www.hhs.gov/hipaa/for-professionals/privacy/special-topics/de-identification/index.html

[54] Malin B A De-identification Strategy Used for Sharing One Data Provider’s Oncology Trials Data through the Project Data Sphere^®^ Repository. (Vol. 19). Project Data Sphere, LLC (2013).

[55] Beaulieu-Jones BK Wu ZS Williams C Greene CS Privacy-preserving generative deep neural networks support clinical data sharing. BioRxiv (2017). 10.1101/159756 PMC7041894 31284738

[56] Merck. Merck Set to Join Forces with Project Data Sphere to Pioneer Global Oncology Big Data Alliance. (2017). Available from: https://www.merckgroup.com/en/news/gobda-mou-signing-2017-09-11.html

[57] The European Medicines Agency. (2016). Available from: https://clinicaldata.ema.europa.eu/web/cdp/home

[58] European Medicines Agency policy/0070 on publication of clinical data for medicinal products for human use. European Medicines Agency. London (2014). 1–22 p. Available from: http://www.ema.europa.eu/docs/en_GB/document_library/Other/2014/10/WC500174796.pdf

[59] Jonker DJ O’Callaghan CJ Karapetis CS Zalcberg JR Tu D Au HJ Cetuximab for the treatment of colorectal cancer. N Engl J Med (2007) 357:2040–8. 10.1056/NEJMoa071834 18003960

[60] Ciardiello F Tortora G EGFR antagonists in cancer treatment. N Engl J Med (2008) 358:1160–74. 10.1056/NEJMra0707704 18337605

[61] De Roock W Claes B Bernasconi D De Schutter J Biesmans B Fountzilas G Effects of KRAS, BRAF, NRAS, and PIK3CA mutations on the efficacy of cetuximab plus chemotherapy in chemotherapy-refractory metastatic colorectal cancer: a retrospective consortium analysis. Lancet Oncol (2010) 11:753–62. 10.1016/S1470-2045(10)70130-3 20619739

[62] Douillard JY Oliner KS Siena S Tabernero J Burkes R Barugel M Panitumumab-FOLFOX4 treatment and RAS mutations in colorectal cancer. N Engl J Med (2013) 369:1023–34. 10.1056/NEJMoa1305275 24024839

[63] ICGC. Submitting Raw Data to EGA. (2017). Available from: http://docs.icgc.org/submission/guide/overview/submitting-raw-data-ega/

[64] Mailman MD Feolo M Jin Y Kimura M Tryka K Bagoutdinov R The NCBI dbGaP database of genotypes and phenotypes. Nat Genet (2007) 39:1181–6. 10.1038/ng1007-1181 17898773 PMC2031016

[65] Zatloukal K BBMRI business plan v21.1. (2012):1–88. Available from: http://www.bbmri-eric.eu/wp-content/uploads/BBMRI-Business-Plan.pdf

[66] GOV.UK. University and Business Collaboration Agreements: Lambert Toolkit. (2017). Available from: https://www.gov.uk/guidance/university-and-business-collaboration-agreements-lambert-toolkit

[67] Asadullah K Busch A Gottwald M Reinke P Landeck L Industry-academia collaborations for biomarkers. Nat Rev Drug Discov (2015) 14:805–6. 10.1038/nrd4727 26514685

[68] FasterCures. Consortia-Pedia – An In-Depth Look at the Research-by-Consortium Trend in Medical Research and Development. (2015). Available from: http://www.fastercures.org/assets/Uploads/Consortia-pedia.pdf

[69] European Commission DG Research. Stratification Biomarkers in Personalised Medicine. European Commission DG Research (2010). Workshop Minutes.

[70] Stevens H Van Overwalle G Van Looy B Huys I Intellectual property policies in early-phase research in public-private partnerships. Nat Biotechnol (2016) 34:504–10. 10.1038/nbt.3562 27153280

[71] Pharmaceutical Researchers and Manufacturers of America, European Federation of Pharmaceutical Industries and Associations. Principles for Responsible Clinical Trial Data Sharing. (2013).

[72] The YODA Project. Johnson & Johnson. (2016). Available from: http://yoda.yale.edu/johnson-johnson

[73] Pencina MJ Louzao DM McCourt BJ Adams MR Tayyabkhan RH Ronco P Supporting open access to clinical trial data for researchers: the Duke Clinical Research Institute-Bristol-Myers Squibb Supporting Open Access to Researchers Initiative. Am Heart J (2016) 172:64–9. 10.1016/j.ahj.2015.11.002 26856217

[74] Clinical Study Data Request Site. Sponsor Specific Information GSK. (2016). Available from: https://www.clinicalstudydatarequest.com/Study-Sponsors-GSK-Details.aspx

[75] Council of Europe. Recommendation CM/Rec(2016)6 of the Committee of Ministers to Member States on Research on Biological Materials of Human Origin (Adopted by the Committee of Ministers on 11 May 2016 at the 1256th Meeting of the Ministers’ Deputies). Council of Europe (2016).

[76] Verlinden M Nys H Ectors N Huys I. Access to biobanks: harmonization across biobank initiatives. Biopreserv Biobank (2014) 12:415–22. 10.1089/bio.2014.0034 25496154

[77] The 100,000 Genomes Project Protocol v4. Genomics England (2017). Available from: https://doi.org/10.6084/m9.figshare.4530893.v4

[78] Peplow M The 100,000 genomes project. BMJ (2016) 1757:i1757 10.1136/bmj.i1757 27075170

[79] Sargent DJ Buyse M Matheson A Goldberg RM de Gramont A The ARCAD clinical trials program: an update and invitation. Oncologist (2012) 17:188–91. 10.1634/theoncologist.2011-0332 22302228 PMC3286167

[80] De Gramont A Haller DG Sargent DJ Tabernero J Matheson A Schilsky RL Toward efficient trials in colorectal cancer: the ARCAD clinical trials program. J Clin Oncol (2010) 28:527–30. 10.1200/JCO.2009.25.2544 19841311

[81] Beacon Network. (2017). Available from: https://beacon-network.org//#/

[82] PerMed2020. SRIA – Strategic Research and Innovation Agenda – Shaping Europe’s Vision for Personalised Medicine. IC PerMed of the European Commission (2015).

[83] Kaye J Curren L Anderson N Edwards K Fullerton SM Kanellopoulou N From patients to partners: participant-centric initiatives in biomedical research. Nat Rev Genet (2012) 13:371–6. 10.1038/nrg3218 22473380 PMC3806497

[84] Kaye J Whitley EA Lund D Morrison M Teare H Melham K. Dynamic consent: a patient interface for twenty-first century research networks. Eur J Hum Genet (2015) 23:141–6. 10.1038/ejhg.2014.71 24801761 PMC4130658

[85] Patients’ Tumor Bank of Hope (PATH) Biobank. (2017). Available from: http://www.path-biobank.org/index.php/en/

[86] Mitchell D Geissler J Parry-Jones A Keulen H Schmitt DC Vavassori R Biobanking from the patient perspective. Res Involv Engagem (2015) 1:4. 10.1186/s40900-015-0001-z 29062493 PMC5598087

[87] Wilkinson MD Dumontier M Aalbersberg IJ Appleton G Axton M Baak A The FAIR Guiding Principles for scientific data management and stewardship. Sci Data (2016) 3:160018. 10.1038/sdata.2016.18 26978244 PMC4792175

[88] Bierer BE Crosas M Pierce HH Data authorship as an incentive to data sharing. N Engl J Med (2017) 376:1684–7. 10.1056/NEJMsb1616595 28402238

[89] Regulation (EU) 2016/679 of the European Parliament and of the Council of 27 April 2016 on the Protection of Natural Persons with Regard to the Processing of Personal Data and on the Free Movement of Such Data, and Repealing Directive 95/46/EC (General Data Protection Regulation). Available from: http://eur-lex.europa.eu/legal-content/EN/TXT/PDF/?uri=CELEX:32016R0679&from=EN

[90] Budin-Ljøsne I Teare HJ Kaye J Beck S Bentzen HB Caenazzo L Dynamic consent: a potential solution to some of the challenges of modern biomedical research. BMC Med Ethics (2017) 18:4. 10.1186/s12910-016-0162-9 28122615 PMC5264333

[91] Scannell JW Blanckley A Boldon H Warrington B Diagnosing the decline in pharmaceutical R&D efficiency. Nat Rev Drug Discov (2012) 11:191–200. 10.1038/nrd3681 22378269

[92] Forda SR Bergström R Chlebus M Barker R Andersen PH Priorities for improving drug research, development and regulation. Nat Rev Drug Discov (2013) 12:247–8. 10.1038/nrd3981 23535921

[93] Hollingsworth SJ Biankin AV. The challenges of precision oncology drug development and implementation. Public Health Genomics (2015) 18:338–48. 10.1159/000441557 26555355

